# DNA methylome profiling of human tissues identifies global and tissue-specific methylation patterns

**DOI:** 10.1186/gb-2014-15-4-r54

**Published:** 2014-04-01

**Authors:** Kaie Lokk, Vijayachitra Modhukur, Balaji Rajashekar, Kaspar Märtens, Reedik Mägi, Raivo Kolde, Marina Koltšina, Torbjörn K Nilsson, Jaak Vilo, Andres Salumets, Neeme Tõnisson

**Affiliations:** 1 Institute of Molecular and Cell Biology, University of Tartu, Tartu, Estonia; 2 Department of Genetics, United Laboratories, Tartu University Hospital, Tartu, Estonia; 3 Estonian Biocentre, Tartu, Estonia; 4 Institute of Computer Science, University of Tartu, Tartu, Estonia; 5 Estonian Genome Center, University of Tartu, Tartu, Estonia; 6 Department of Medical Biosciences, Clinical Chemistry, Umeå University, Umeå, Sweden; 7 Competence Centre on Reproductive Medicine and Biology, Tartu, Estonia; 8 Department of Obstetrics and Gynecology, University of Tartu, Tartu, Estonia; 9 Institute of Bio- and Translational Medicine, University of Tartu, Tartu, Estonia

## Abstract

**Background:**

DNA epigenetic modifications, such as methylation, are important regulators of tissue differentiation, contributing to processes of both development and cancer. Profiling the tissue-specific DNA methylome patterns will provide novel insights into normal and pathogenic mechanisms, as well as help in future epigenetic therapies. In this study, 17 somatic tissues from four autopsied humans were subjected to functional genome analysis using the Illumina Infinium HumanMethylation450 BeadChip, covering 486 428 CpG sites.

**Results:**

Only 2% of the CpGs analyzed are hypermethylated in all 17 tissue specimens; these permanently methylated CpG sites are located predominantly in gene-body regions. In contrast, 15% of the CpGs are hypomethylated in all specimens and are primarily located in regions proximal to transcription start sites. A vast number of tissue-specific differentially methylated regions are identified and considered likely mediators of tissue-specific gene regulatory mechanisms since the hypomethylated regions are closely related to known functions of the corresponding tissue. Finally, a clear inverse correlation is observed between promoter methylation within CpG islands and gene expression data obtained from publicly available databases.

**Conclusions:**

This genome-wide methylation profiling study identified tissue-specific differentially methylated regions in 17 human somatic tissues. Many of the genes corresponding to these differentially methylated regions contribute to tissue-specific functions. Future studies may use these data as a reference to identify markers of perturbed differentiation and disease-related pathogenic mechanisms.

## Background

DNA methylation is the most extensively studied epigenetic modification of mammalian DNA [1]. DNA methylation of cytosine residues mainly occurs in CpG sequences and has been characterized as an important regulatory mechanism of genome function, having been implicated as a crucial mediator of embryonic development, transcription, chromosomal stability, imprinting, and X-chromosome inactivation [2]. The DNA methylation profile itself is not static and subject to dynamic changes induced by age-related factors [3], environmental factors [4], nutritional factors, and pathogenic factors, such as viruses [5,6].

Many previous studies have investigated the DNA methylation profiles of various human tissues and conditions. These studies have mainly relied on high-throughput DNA detection methods and sequencing technologies, such as the HumanMethylation450 BeadChip [7], HumanMethylation27 BeadChip [8] and GoldenGate Methylation Cancer Panel I [9-11] arrays (Illumina Inc., San Diego, CA, USA), or microarrays in combination with methylated DNA enrichment by immunoprecipitation [12]. Some previous studies have concentrated on CpG islands in promoter regions and characterized for their role in changes to the gene’s expression [8,10], but increasingly more studies are identifying tissue-specific differentially methylated regions (tDMRs) in the gene body regions [12,13]. Although all the previous studies have enabled a broader view of the genome-wide DNA methylation patterns, there still remain questions to be answered, for example, how the tDMRs are being established and what are the functions of gene-body tDMRs. Determining the human tDMR profile will not only provide important insights into the normal processes of tissue-specific differentiation but may identify markers of pathogenic processes, such as cancer.

In this study, we analyzed the tissue-specific DNA methylome using a panel of 17 somatic tissues obtained from four autopsied individuals. The expanded Illumina Infinium HumanMethylation450 BeadChip was used to interrogate 486 428 CpG sites in the human genome; this advanced platform boasts unbiased coverage of gene and CpG island (CGI) regions reaching up to 99% and 96%, respectively, as well as CpG island shores (2 kb regions upstream and downstream of the CpG islands) and shelves (2 kb regions upstream and downstream of the CpG island shores) to reveal a genome-wide methylation profile [14].

Our aim was to describe the general patterns of globally conserved and tissue-specific DNA methylation with functional consequences in gene regulation. Using the high-density microarray allowed nearby CpG sites with similar patterns to be grouped together so as to identify broader regions of tDMRs and improve the statistical power of the analysis. Our results reveal tissue-specific methylation patterns beyond the well-studied promoter areas, identifying tDMRs in gene body areas and showing these regions to be more likely related to tissue-specific functions. Collectively, these data represent novel insight into the regulatory role of tissue-specific DNA methylation.

## Results and discussion

### Methylome profiling across 17 somatic tissues

Tissue-specific DNA methylation patterns were studied in the following 17 somatic tissues: abdominal and subcutaneous adipose tissue, bone, joint cartilage, yellow and red bone marrow, coronary and splenic artery, abdominal and thoracic aorta, gastric mucosa, lymph node, tonsils, bladder, gall bladder, medulla oblongata, and ischiatic nerve. Samples of each of these postmortem specimens were obtained from four individuals upon autopsy, except in the case of one individual (Identification No. BM419/4) for whom the yellow bone marrow and joint cartilage tissues were not available. The causes of death included: intracerebral hemorrhage (BM419/4; female, 60 years old), heart attack with acute cardiac insufficiency (KA522; male, 53 years old), heart attack (KT538; male, 40 years old), and intracerebral hemorrhage (SJ600-5; male, 54 years old).

Genomic DNA was extracted from each tissue, treated with sodium bisulfite, and subjected to analysis via the Illumina Infinium HumanMethylation450 BeadChip. The methylation levels of CpGs were described as beta values (0 to 1) representing the calculated level of methylation (0% to 100%). We had two technical and two biological replicates processed by chip technique. The Pearson correlation coefficients (PCCs) were >0.99 for all the replicates, confirming a good level of reproducibility for the chip process and indicating that the observed differential methylation between the studied tissues represented true biological differences.

Several of the observed DNA methylation differences were selected for verification by conventional Sanger dideoxy sequencing. More specifically, the detected CpG methylation levels of 17 genes encompassing 36 CpGs, including 0% (*n* = 1) and 100% (*n* = 2) methylated sites, and of 14 genes with tDMRs, were confirmed by bisulfite sequencing. The BeadChip data strongly correlated with the Sanger sequencing data (mean PCC: 0.93, PCC range: 0.78 to 0.98; Additional file 1). Methylation levels of CpGs adjacent to those present on the BeadChip were also strongly correlated with the Sanger sequencing data (mean PCC 0.95, PCC range: 0.72 to 1.00). Most of the CpGs detected were clustered together, but some CpGs with similar methylation levels and corresponding to a known gene or regulatory region were located >200 bp apart (data not shown). Thus, uniform CpG methylation may involve longer distances for tissue-specific regulatory mechanisms.

**Figure 1:**
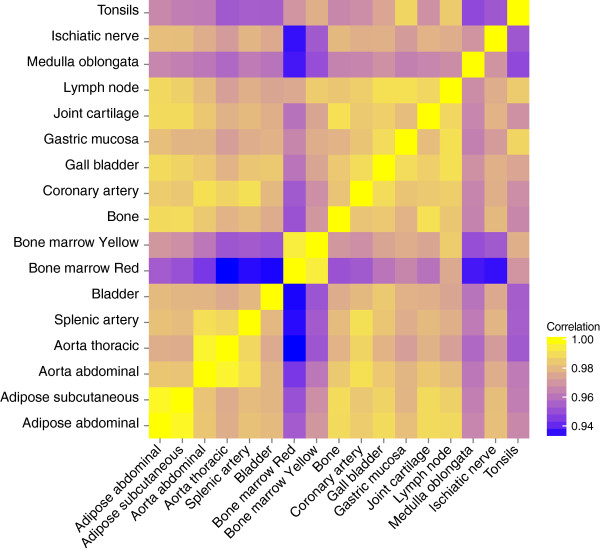
**Correlation of methylation intensities between tissues.** The mean methylation levels of each CpG site within different specimens of the same tissue were compared and the PCC was calculated. The correlation matrix of different tissues is shown; the tissues appear to show a similar trend, for which the highest correlations occur between functionally similar tissues.

**Figure 2:**
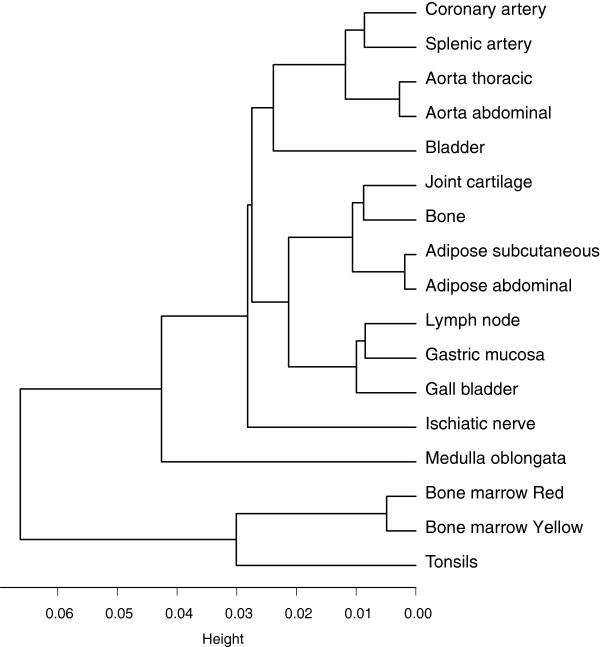
**Hierarchical clustering of the 17 tissues studied.** Hierarchical clustering analysis was performed using the hclust command in R. All of the samples were merged according to their corresponding tissues, which resulted in a matrix of the mean beta values for all of the CpG sites detected in the 17 total tissues. The clustering tree was generated using the complete method. The tree shows strong correlation between similar tissue types.

Comparative analysis of the DNA methylation patterns between tissues was carried out to determine a general relatedness profile. The methylation patterns were found to be well conserved between the 17 various tissues that were studied (Figure 1). The lowest correlations were found for red bone marrow *versus* thoracic and splenic artery, *versus* bladder, and *versus* medulla oblongata and ischiatic nerve (PCC: 0.93). The highest correlations were found among functionally similar tissues, such as the different arteries and aortas, red and yellow bone marrow, and bone and joint cartilage (PCC: ≥0.99). Hierarchical clustering of the methylation profiles of the 17 studied tissues showed that most of the similar tissues (for example, aortas and arteries) co-clustered (Figure 2); the strong correlations indicated between similar tissues suggested the presence of tissue-specific methylation profiles.

### Genome-wide DNA methylation patterns

**Figure 3:**
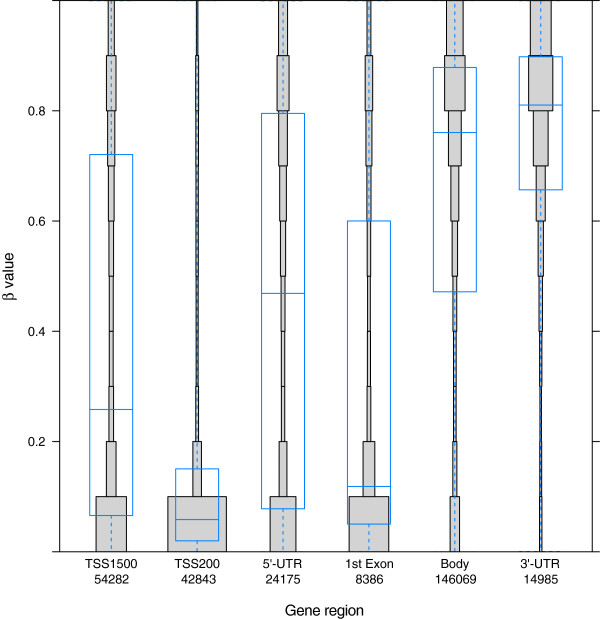
**DNA methylation in specific gene regions.** Distribution of DNA methylation in specific gene regions is shown. Each gene region is further divided into bins that correspond to beta values with 0.1 intervals. The area of each bin corresponds to total number of CpGs. The overall distribution and the mean of beta value of the CpGs in each gene region are shown as a box plot. The most unmethylated regions are associated with promoter sequences (TSS1500, TSS200, and 5′-UTR) and the first exon, while the most methylated regions are in the gene body and 3′-UTR. The numbers on the x-axis correspond to total number of CpGs in each gene region; also the x-axis shows different gene regions, and the y-axis shows the beta values.

**Figure 4:**
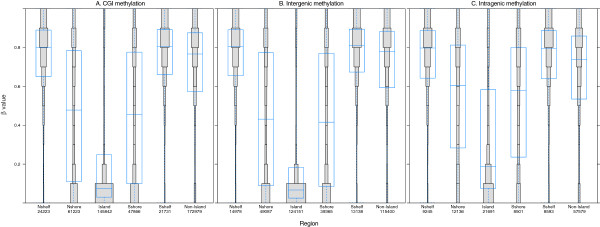
**CGI methylation in different genomic regions.** Distribution of DNA methylation in specific gene regions is shown. Each gene region is further divided into bins that correspond to beta values with 0.1 intervals. The area of each bin corresponds to total number of CpGs. The overall distribution and the mean of beta value of the CpGs in each gene region are shown as a box plot. **(A)** The distribution of DNA methylation in CGI and non-CGI regions shows that the CGI itself is largely unmethylated and that the shores and shelves are methylated. **(B, C)** The distribution of CGI and non-CGI DNA methylation in intergenic **(B)** and intragenic **(C)** regions. **(A-C)** The numbers on the x-axis correspond to total number of CpGs in each gene region; also the x-axis shows different genomic regions, and the y-axis shows the beta values.

Investigation of the global distribution of CpGs in somatic tissues according to the methylation status revealed that a large portion of the detected CpGs are either unmethylated (0%) or fully methylated (100%) (Additional file 2). Considering the collected data for all 17 tissues indicated that only 2.2% of all the CpGs (10,707 CpGs representing 4,416 genes) were hypermethylated in all of the samples (beta values >0.9). These invariably methylated CpGs were mostly located in gene bodies, in the 3’-untranslated regions (UTRs) (66.8%, 7,150 CpGs; Figure 3) or in non-CGIs (77.4%, 8,287 CpGs; Figure 4A) (Fisher’s exact test, *P* <2.2 × 10^-16^). Thus, DNA methylation appears to be more prominent in the areas where CpG density is low and transcription is not usually initiated.

On the other hand, 14.9% of CpGs (72,444 CpGs representing 12,604 genes) were hypomethylated in all of the samples (beta values <0.1). These invariably hypomethylated CpGs were mostly located in gene promoter areas (73.2%, 53,057 CpGs), including the sequence region from -200 to -1,500 nt upstream of the transcription start site (TSS1500), the region from -200 nt upstream to the TSS itself (TSS200), and the region from the 5’-UTR through the first exon (Figure 3). In addition, the hypomethylated CpGs were found in CGI regions (73.0%, 52,862 CpGs; Figure 4A) (Fisher’s exact test, *P* <2.2 × 10^-16^). These findings are consistent with the general consensus that gene promoter areas and CGI regions of actively transcribed genes are largely unmethylated so as to be accessible to transcription factors.

Gene ontology (GO) analysis with the Database for Annotation, Visualization and Integrated Discovery (DAVID [15]) revealed that many of the genes showing hypermethylation of their CGI-promoter regions had functions related to the reproductive system; in contrast, many of the genes showing hypomethylation of their CGI-promoter regions had functions associated with housekeeping processes, including RNA processing and cell cycle. When our data of hypomethylated CGI-promoter regions were compared to the housekeeping genes identified by expression profiling in a previous study by Chang et al. [16], we found a 93.0% consensus.

We also found that the DNA methylation pattern of a single gene varies between gene regions; for example, compared to the gene body, the TSS1500, TSS200, 5’-UTR, and first exon showed lower average methylation (Figure 3). These data agree with those from previous studies and in line with the notion that promoter areas of housekeeping genes would be accessible to support active transcription [17].

### Comparison of DNA methylation in CGI and non-island regions

It is well recognized that DNA methylation patterns can differ significantly across the different regions of CGIs, with methylation levels increasing at the boundaries. In our study, the highest levels of methylation were found in the CGI shelves and shores (Figure 4A). These results are in agreement with those of previous studies [17,18], in which the majority of CGIs were shown to be unmethylated. Also, in our study, the CGI methylation patterns were found to be largely consistent within intergenic regions and in genes (Figure 4B and 4C). It is possible that maintaining an unmethylated state in a CGI may serve to protect against mutation by spontaneous deamination of methylated cytosines [19].

**Figure 5:**
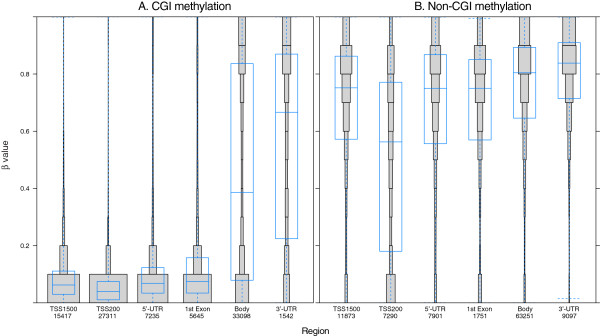
**DNA methylation distribution in CGI and non-CGI regions.** Distribution of DNA methylation in specific gene regions is shown. Each gene region is further divided into bins that correspond to beta values with 0.1 intervals. The area of each bin corresponds to total number of CpGs. The overall distribution and the mean of beta value of the CpGs in each gene region are shown as a box plot. **(A)** DNA methylation is low in promoter areas, but high in non-CGI regions of all gene areas **(B)**. **(A-B)** The numbers on the x-axis correspond to total number of CpGs in each gene region; also the x-axis shows different genomic regions, and the y-axis shows the beta values.

Comparison of CGI methylation patterns across different parts of individual genes revealed that the promoter areas (TSS1500, TSS200, and 5’-UTR) and the first exon were almost exclusively unmethylated; however, variable CGI methylation levels were found in the gene body and 3’-UTR (Figure 5A). This pattern has been observed by other studies, as well [12,18]. In contrast, the CpGs found in non-CGI regions were found to be mostly methylated, and showed little variation across the different parts of the individual genes (Figure 5B). Comparative analysis of the methylation patterns in CGI shores and shelves in different gene regions revealed that the CGI and CGI shore regions are generally similar, but the CpGs in the shelves are nearly fully methylated (data not shown).

### Tissue-specific differentially methylated regions

Next, the regions with distinctive methylation patterns in certain tissues were analyzed in detail. We applied an algorithm to identify statistically significant differential methylation existing between two sets of samples in three or more consecutive CpG probes. This new method is based on fitting ANOVA models in moving windows of different lengths, encompassing up to 50 probes. The optimal region boundaries were selected according to the minimum description length (MDL) principle. As a result, every region consists of probes that have similar methylation patterns. As HumanMethylation450 BeadChip is focused more on the genes and promoter areas, this robust approach finds more likely regions with a higher CpG probe density.

We used this method to detect tDMRs between one tissue of interest and all other tissues under study. For this analysis, the data from some of the tissues used in this study were combined to correct for the high level of functional similarity that existed between them; specifically, abdominal and subcutaneous adipose tissues were processed together, as were the thoracic and abdominal aorta, coronary and splenic artery, joint cartilage and bone, and red and yellow bone marrow.

The numbers of different tDMR CpG blocks were found to vary greatly between tissues with different functions (Table 1). The highest number of hypermethylated tDMRs was found in tonsils, followed by medulla oblongata and aortas (abdominal and thoracic). The lowest number of hypermethylated tDMRs was found in lymph nodes. For hypomethylated tDMRs, large numbers were found in bone marrow (red and yellow), aortas (abdominal and thoracic), and ischiatic nerve, while the lowest number was found in the lymph nodes. Of the total 14,441 tDMRs identified (Additional file 3), 11,242 (77.8%) mapped to genes. Among those, 41.7% (4,688) were in gene promoter areas with only 36.5% in CGIs (Fisher’s exact test, *P* <2.2 × 10^-16^), and more than one-half (58.3%, 6,554) in gene body regions with 44.1% in CGIs (Fisher’s exact test, *P* <2.2 × 10^-16^). The fact that over a half of tDMRs were located in gene bodies and not in promoter areas is intriguing because methylation within a gene body may indicate the presence of alternative promoters [20]. Among the intergenic tDMRs, 45.8% co-localized with CGIs (Fisher’s exact test, *P* = 0.0003), intergenic regions might act as regulators, being either enhancers or silencers and contributing with these mechanisms into maintenance of tissue-specific gene expression. These results are in line with those of previous studies, which have shown that tDMRs exist across a range of CpG densities, while tDMRs in promoter areas are largely located in non-CGI regions [7,11,12,18].

**Table 1:** tDMR data summary

**Tissue**	**Hypermethylated blocks**	**Hypermethylated blocks with gene annotation**	**Hypomethylated blocks**	**Hypomethylated blocks with gene annotation**
Adipose (subcutaneous, abdominal)	84	65	376	301
Artery (coronary, splenic)	380	280	283	219
Bone, joint cartilage	129	73	168	104
Bone marrow (red, yellow)	175	150	1,300	1,028
Gastric mucosa	74	54	26	22
Lymph node	56	42	5	3
Tonsils	4,983	3,893	1,072	924
Bladder	379	274	752	566
Gall bladder	65	47	93	66
Aorta (thoracic, abdominal)	628	453	1120	888
Medulla oblongata	651	495	349	278
Ischiatic nerve	203	156	1,090	861

In order to study which regions are the most variable between tissues, we compared the proportion of variance explained by the tissues between different gene regions and between CpG islands, shores, and shelves. Additional file 4A and B show the distributions of the R squared statistic, respectively. We can see that in gene body, 3’-UTR and the sites that are not related to genes, exist larger differences between the tissues. But in the gene promoter areas the methylation patterns of tissues are much more similar. This is supported also from the results above, that large number of tDMRs were found within gene body regions. Also, in CpG islands different tissues are more similar than in shores, shelves, and non-island sites.

To characterize the function of genes related to the detected tDMRs, we again performed GO analysis using the DAVID database. We have used a custom background in the GO enrichment analysis, which contains all the genes that were found as tDMRs. This should take in account the distribution of CpG probes on microarray. As shown in Table 2, those genes showing hypomethylation in certain tissues are frequently associated with a tissue-specific function. For example, the hypomethylated genes detected in arteries (including *COL18A1*, *EPAS1*, *ENPEP*, *ANGPT2*, and *APOLD1*) are characterized as mediators of blood vessel development and morphogenesis, while those detected in tonsils (including *LAX1*, *TNFSF14*, *LCK*, and *RHOH*) are involved in immune response and leukocyte activation.

**Table 2:** GO analysis with hypomethylated tDMRs

**Tissue**	**GO term**	**Genes (*n*)**	***P *** **value**
Adipose tissue (abdominal, subcutaneous)	Lipid homeostasis	5	0.0096
	White fat cell differentiation	3	0.0172
	Fat cell differentiation	4	0.0532
Artery (coronary, splenic)	Blood vessel morphogenesis	12	3.24E-04
	Angiogenesis	10	4.25E-04
	Blood vessel development	13	4.60E-04
Aorta (thoracic, abdominal)	Cardiac muscle tissue development	11	5.91E-04
	Muscle organ development	24	8.21E-04
	Striated muscle tissue development	16	9.72E-04
Bone, joint cartilage	Chondrocyte differentiation	3	0.0067
	Cartilage development	4	0.0253
	Skeletal system development	7	0.0553
Bone marrow (red, yellow)	Cell activation	41	5.07E-07
	Leukocyte activation	33	7.98E-06
	Immune response	62	1.97E-05
Lymph node	-		
Tonsils	Immune response	59	7.00E-06
	Regulation of T cell activation	17	4.38E-05
	Defense response	50	7.22E-05
Gastric mucosa	Regulation of pH	2	0.0530
	Monovalent inorganic cation homeostasis	2	0.0677
Bladder	Muscle contraction	14	0.0034
	Excretion	7	0.0266
	Secretion	17	0.0379
Gall bladder	Negative regulation of granulocyte differentiation	2	0.0417
	Negative regulation of immune system process	3	0.0496
	Regulation of granulocyte differentiation	2	0.0519
Medulla oblongata	Homophilic cell adhesion	15	8.78E-06
	Cell-cell adhesion	18	5.00E-04
	Cell adhesion	25	0.0151
Ischiatic nerve	Filopodium assembly	5	0.0023
	Regulation of action potential in neuron	10	0.0036
	Negative regulation of neurogenesis	7	0.0074

In agreement with previous results, none of the genes showing hypermethylation in specific tissues were associated with tissue-specific biological processes (Additional file 5) [8]. Thus, our results, along with those from earlier studies, strongly support the hypothesis that hypomethylation, and not hypermethylation of genes, is more likely to be associated with the tissue-specific functions.

### Inter-individual methylation variation

**Figure 6:**
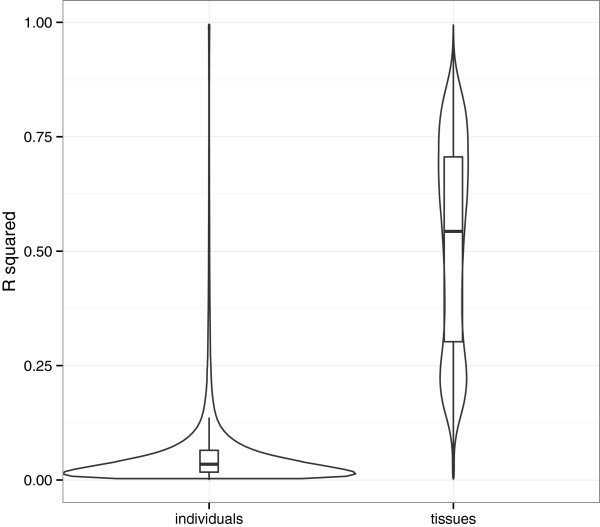
**Variance explained by the tissues and individuals.** Figure is showing the distribution of the R squared statistic obtained for each CpG site. It is clearly visible, that variance explained by the individuals is insignificant - on average individuals explain only 6.4% of the variance whereas tissues explain 51.2%.

We analyzed the rate of inter-individual variation in order to understand whether individuals or tissues are explaining most of the variability between samples. We compared the proportion of variance of the beta values explained by the individuals and the proportion of variance explained by the tissues. Figure 6 shows the distribution of the R squared statistic obtained for each CpG site. On average, we can see that individuals explain only 6.4% of the variance whereas tissues explain 51.2%, showing that although the variance between individuals exist, it is really insignificant.

The hierarchical clustering (Additional file 6) of all the samples studied is also showing that the similarity between different tissues was higher than between individuals, as tissues are mostly clustering together. As the number of individuals under investigation was relatively small (*n* = 4; one woman and three men) and also the majority of the phenotypic data was lacking, we did not find relevant to analyse the inter-individual methylation variation in detail. Furthermore, it is explaining only subset of variance between samples.

### Relation between gene expression and global DNA methylation

To further investigate the role of DNA methylation in regulation of gene expression, we compared the detected methylation patterns with publicly available gene expression data (Gene Expression Omnibus (GEO) and ArrayExpress databases). Only tissues with gene expression data obtained using a single platform (Human Genome U133A arrays; Affymetrix, Santa Clara, CA, USA) were selected to decrease the impact of potential confounding factors. As a result, correlations of gene expression levels were carried out for eight of the 17 tissues used in the original analysis: aorta, bladder, bone, bone marrow, coronary artery, lymph node, medulla oblongata, and tonsils (Additional file 7).

The method by which the global methylation data were correlated with the gene expression data relied on averaging beta values across the comprehensive gene panel. The PCCs were calculated for 10,120 genes across the eight tissues (Table 3) and revealed a slight bias towards negatively-correlated genes’ expression (5,710 *vs*. positively-correlated: 4,410 genes). In addition, nearly twice as many genes showed a strong inverse correlation (1,713 genes, PCCs: <-0.5) than those showing a strong positive correlation (1,090 genes, PCCs: >0.5) (Fisher’s exact test, *P* <2.2 × 10^-16^).

**Table 3:** Gene expression and methylation correlation

**Gene region**	**Neg PCCs**^**a**^	**Pos PCCs**^**b**^	**PCCs < -0.5**^**c**^	**PCCs >0.5**^**d**^	**Total**^**e**^
Global	5,710	4,410	1,713	1,090	10,120
	56.42%	43.58%	16.93%	10.77%	
Promoter + CGI	3,048	2,325	618	567	5,373
	56.73%	43.27%	11.50%	10.55%	
TSS1500	4,175	4,581	958	1,064	8,756
	47.68%	52.32%	10.94%	12.15%	
TSS200	4,157	3,199	979	747	7,356
	56.51%	43.49%	13.31%	10.15%	
5’UTR	1,500	1,604	382	442	3,104
	48.32%	51.68%	12.31%	14.24%	
1st exon	1,330	1,409	272	357	2,739
	48.56%	51.44%	9.93%	13.03%	
Body	5,156	4,620	1,573	1,185	9,776
	52.74%	47.26%	16.09%	12.12%	
3’UTR	3,302	3,494	903	854	6,796
	48.59%	51.41%	13.29%	12.57%	
Shores	4,534	3,365	1,362	792	7,899
	57.40%	42.60%	17.24%	10.03%	
Shelves	2,342	2,201	718	576	4,543
	51.55%	48.45%	15.80%	12.68%	

^a^Negative Pearson Correlation coefficients (PCCs).

^b^Positive PCCs.

^c^Strong negative PCCs, smaller than -0.5.

^d^Strong positive PCCs, larger than 0.5.

^e^Total number of tDMRs in named category.

When analyzing the correlation of global methylation data with different gene regions, the number of negatively-correlated genes in CGI-promoter areas (56.7%) was found to be roughly the same as that in gene bodies (52.7%). Slightly more genes showed a strong inverse correlation than those showing a strong positive correlation, both for methylation located within the promoter area and the gene body (11.5% and 10.6% in promoter-CGI; 16.1% and 12.1% in gene body, respectively) (Fisher’s exact test, *P* = 0.005).

**Figure 7:**
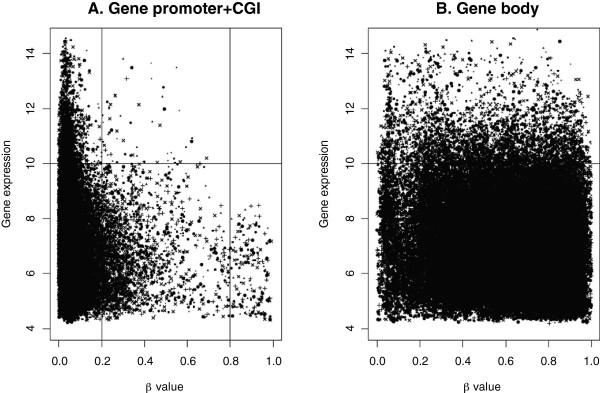
**DNA methylation and gene expression correlation in CGI-promoter regions and the gene body. (A)** Correlation analysis of CGI-promoter methylation and gene expression show that genes with low expression have high methylation. **(B)** Gene body methylation and gene expression are not correlated. **(A, B)** The x-axis shows DNA methylation beta values, and the y-axis shows gene expression values. The different tissues studied are represented by the following symbols: aortas (•), coronary artery (●), bladder (), bone (), bone marrows (), lymph node (), medulla oblongata (+), and tonsils (×).

When DNA methylation and gene expression values are similar among a set of various tissues, correlation analysis may be insufficient. To correct for this possibility in our dataset, the methylation and gene expression data were plotted onto a single figure so that the integrity of the correlation between CGI-promoter and gene body areas could be further assessed (Figure 7). In the CGI-promoter areas, high levels of methylation were found to be associated with lower gene expression and low levels of methylation were associated with higher, and varying, levels of gene expression. However, the same relationship was not observed for the data related to gene bodies; the fully methylated and unmethylated genes showed a similar varying trend in their gene expression levels.

Our analysis of highly methylated promoters suggested a possible link between the promoter methylation and suppressed gene expression. Similar to our findings, previous studies have reported that genes with unmethylated promoters show variable levels of transcription activity [12,18]. Our analysis of methylation in gene bodies revealed no clear relationship with mRNA expression levels, although previous studies have reported either positive correlation with gene expression [12,21] or bell-shaped correlation patterns [22]. Many genes harbor several alternative TSSs, which are located throughout the gene body and yield different splice isoforms. Methylation of such yet unrecognized sites might confound a correlation analysis of gene body methylation and gene expression.

### Gene expression and methylation in tDMRs

The correlation analysis of tissue-specific methylation with gene expression was carried out by averaging all of the CpG beta values within the tDMRs. Collectively, there were more negative than positive correlation coefficients (63.2%, 2,288 *vs*. 36.8%, 1,332; Table 4), as expected. In addition, strongly negative PCCs prevailed over the strongly positive PCCs (20.7%, 749 *vs*. 10.3%, 372, respectively) (Fisher’s exact test, *P* <2.2 × 10^-16^).

**Table 4:** Gene expression and methylation correlation in tDMRs

**Gene region**	**Neg PCCs**^**a**^	**Pos PCCs**^**b**^	**PCCs < -0.5**^**c**^	**PCCs >0.5**^**d**^	**Total**^**e**^
Global	2,288	1,332	749	372	3,620
	63.2%	36.8%	20.7%	10.3%	
Promoter + CGI	489	134	76	37	623
	78.5%	21.5%	12.2%	5.9%	
5’UTR	264	279	57	77	543
	48.6%	51.4%	10.5%	14.2%	
1st exon	212	217	41	61	429
	49.4%	50.6%	9.6%	14.2%	
Body	1,148	737	377	224	1,885
	60.9%	39.1%	20.0%	11.9%	
3’UTR	190	209	51	62	399
	47.6%	52.4%	12.8%	15.5%	
Shores	1,275	745	423	202	2,020
	63.1%	36.9%	20.9%	10.0%	
Shelves	445	269	169	82	714
	62.3%	37.7%	23.7%	11.5%	

^a^Negative Pearson Correlation coefficients (PCCs).

^b^Positive PCCs.

^c^Strong negative PCCs, smaller than -0.5.

^d^Strong positive PCCs, larger than 0.5.

^e^Total number of tDMRs in named category.

Our finding of relatively more negative correlations in the gene bodies (60.9%, 1,148) was slightly unexpected, because gene body methylation is not usually related to low expression. However, our finding of a high number of inversely correlated CpG sites in CGI-promoter regions (78.5%, 489) and finding that genes with highly methylated promoter areas were not highly expressed suggest that methylation in the promoter area corresponds to gene expression changes (Additional file 8).

## Conclusions

In this study, we analyzed the genome-wide DNA methylation profiles of human somatic tissues. Although the number of analyzed individuals was limited, the analysis was sufficient to provide DNA methylation distribution patterns across different genomic regions that were largely in agreement with patterns previously observed by similar studies. Moreover, our results and their validation by external datasets revealed a clear correlation between DNA methylation in the gene promoter areas and the gene expression. Meanwhile, our analysis of methylation in gene bodies did not reveal positive [12,21] or bell-shaped [22] correlation patterns with mRNA expression levels, as it is suggested before.

The methylome data alone was sufficient for correctly distinguishing, through hierarchical clustering, between all the 17 tissues studied, collectively demonstrating that tissues are characterized by distinctive methylation patterns that reflect their tissue-specific functions. We were also able to show that the variance explained by tissues is much higher than the variance explained by individuals. As a result of differentially methylated tissue-specific regions analysis, we identified a large number of tDMRs, which were enriched for genes that are closely related to the functions of particular tissue type. Moreover, hypomethylation, and not hypermethylation, was more likely to be associated with the tissue-specific functions.

Our study also provoked the question, of how tDMRs mechanistically contribute to the tissue-specific functions, especially for the numerous methylation regions that were found in gene body areas. In addition, the observation that the methylation in the gene body areas had also high negative correlation with gene expression suggested that gene body tDMRs might be important in establishing the tissue-specific transcription. Still, it remains unclear, however, how the gene body tDMRs may function as regulators of gene expression, and this question should be addressed in the future epigenetic studies.

To our knowledge, this study comprehends methylation data of tissue types that have not been studied yet. The data are publicly available to the research community, as well as the annotated UCSC tracks.

## Materials and methods

### Ethics statement

The Research Ethics Committee of the University of Tartu approved the collection of tissue samples for research (permission no 221/M-18). Written informed consent was obtained from next-of-kin to postmortem individuals in order to collect the tissue panel during the autopsy. The research was carried out according to the World Medical Association Declaration of Helsinki.

### Sample collection and DNA preparation

The 17 postmortem human somatic tissues used in this study were collected at the time of autopsy. All specimens were subjected to autolysis for 4 to 8 h and then snap-frozen at -80°C until use in analysis. DNA was extracted from 25 mg samples of the tissue specimens using the NucleoSpin® Tissue kit (Macherey-Nagel GmbH, Düren, Germany). The DNA yield and purity were determined spectrophotometrically (NanoDrop® ND1000; Thermo Fisher Scientific Inc., Waltham, MA, USA) and by gel electrophoresis, respectively. Bisulfite modification of the genomic DNA samples (600 ng each) was carried out with the EZ DNA Methylation™ kit (Zymo Research, Orange, CA, USA) according to the manufacturer’s protocol.

Controls for unmethylated and methylated DNA were represented, respectively, by whole-genome amplified DNA from subcutaneous adipose tissue (using the GenomiPhi DNA amplification kit; GE Healthcare, Piscataway, NJ, USA) and the universal methylated human DNA standard (Zymo Research). The bisulfite treatment of the control samples was carried out as described above.

### Methylation analysis with illumina infinium HumanMethylation450 BeadChip

DNA methylation analysis of the total 72 tissue samples and controls was performed with the Illumina Infinium HumanMethylation450 BeadChip according to the manufacturer’s standard protocols. This BeadChip contains more than 485,000 methylation sites, covering 99% of RefSeq genes with an average of 17 CpGs per gene distributed across the promoter, 5′-UTR, first exon, gene body, and 3′-UTR regions [14]. In addition, the BeadChip covers 96% of CGI with an average of five CpG sites each, as well as the corresponding shores and shelves. Furthermore, it includes CpGs outside of CGIs, CGIs outside of coding regions, and micro-RNA promoter regions.

### Validation of BeadChip methylation data by Sanger sequencing

Seventeen genes representing 36 CpG sites (including three unmethylated and fully methylated sites, and 14 genes with tDMRs) were selected for analysis. Primers for PCR amplification of the bisulfite-treated DNA were designed using MethPrimer [23] and are listed in Additional file 9. The 20 μL reaction mixes contained 80 mM Tris-HCl (pH 9.4 to 9.5), 20 mM (NH_4_)_2_SO_4_, 0.02% Tween-20 PCR buffer, 3 mM MgCl_2_, 1X Betaine, 0.25 mM dNTP mix, 2 U Smart-*Taq* Hot DNA polymerase (Naxo, Tartu, Estonia), 50 pmol forward primer, 50 pmol reverse primer, and 20 ng bisulfite-treated genomic DNA. The PCR cycling conditions were: 15 min at 95°C for enzyme activation, followed by 17 cycles of 30 s at 95°C, 45 s at 62°C, and 120 s at 72°C, with a final -0.5°C/cycle step-down gradient over 21 cycles of 30 s at 95°C, 30 s at 52°C, and 120 s at 72°C. The sequencing results were analyzed with Mutation Surveyor software (Softgenetics, State College, PA, USA) and the R statistical computing software [24].

### Data normalizing and preprocessing

The raw data were subject to quality control and normalization using the standard protocols suggested for the bioconductor R package minfi [25]. All probes containing single nucleotide polymorphisms (*n* = 65) and CpG sites from the X (*n* = 11 232) and Y (*n* = 416) chromosomes were removed from the analysis, in order to eliminate the effect of sex-specific methylation.

### GO analysis

GO analysis was carried out for the differentially hypomethylated and hypermethylated regions between tissues using DAVID [15,26]). The gene sets that showed hyper- or hypomethylation were searched against a default population background (*Homo sapiens*) and results were matched with GO biological processes (GOTERM BP-FAT). The gene sets obtained from tDMR analysis for each specific tissue were searched against a custom background, which contained all the genes found by tDMR analysis.

### Correlation analysis of DNA methylation with gene expression

Gene expression data were obtained from the GEO [27] and ArrayExpress [28] databases. Eight tissues with data from the Affymetrix Human Genome U133A Array (HG-U133A) were selected for analysis; the accession numbers of the datasets used are listed in Additional file 7. For correlating the global DNA methylation data with gene expression values, the DNA methylation values were averaged across the gene. Gene expression data were normalized and preprocessed according to the robust multi-array average algorithm [29]. All statistical analyses were performed by R statistical computing software.

### Algorithm for identifying tDMRs

An MDL-based method that is similar to the one proposed for finding haplotype blocks was used to identify differentially methylated regions [30]. In principle, we fit the same statistical model by moving windows of 1 to 50 probes in width and calculate the description length statistic. Intuitively, when the same model fits well to several consecutive probes, then one model for all these probes is less costly, in terms of description length, than several separate models. Based on the model fit and its description length, the probes were segmented into regions that, in total, give the MDL.

To identify the tDMRs of the studied tissues, the analysis of variance (ANOVA) model with an MDL framework was used. For each segment, the model was fitted to compare the tissue of interest against all other tissues studied. The tDMRs were identified according to detection by at least three probes and their retaining statistical significance (*P* <0.05) after Bonferroni correction. To help identify regions of realistic length, the search was conducted only in regions where the distance between consecutive probes was less than 3 kb. It has been shown that sequence-specific DNA methylation as a regulatory mechanism works on regions larger than 1,000 base pairs [31]. Also, it has been suggested that long-CGI promoters (>2,000 bp) are preferentially associated with genes that are important in development and tissue-specific gene expression [32]. Additional file 10 shows the correlation between methylation beta values of consecutive probes and how it depends on the distance between these probes. The conservative choice of a 3 kb cutoff was based on this distribution of correlations, because for larger distances the average correlation is only 0.18 whereas for shorter distances it is 0.42. Meanwhile, these blocks are considered as one region only if the methylation dynamics within the region are similar enough (in terms of the MDL). Tissues with a high functional similarity were processed together.

### Data access

The data used in this study has been deposited in NCBI’s Gene Expression Omnibus repository and are accessible through GEO Series, accession number GSE50192. Also, the raw data and some extra figures are available on the website [33].

## Abbreviations

ANOVA: Analysis of variance; CGI: CpG island; GEO: Gene Expression Omnibus; GO: Gene ontology; MDL: Minimum description length; PCC: Pearson correlation coefficient; tDMRs: Tissue-specific differentially methylated regions; TSS: Transcription start site; TSS1500: -200 to -1,500 nt sequence upstream of TSS; TSS200: -200 nt sequence upstream of TSS.

## Competing interests

The authors declare that they have no competing interests.

## Authors’ contributions

KL carried out the studies and drafted the manuscript. VM and BR performed the methylome profiling and gene expression correlation. RM and BR carried out the hierarchical clustering analysis. KM and RK performed the tDMR and inter-individual analysis. KL made the gene ontology analysis. MK carried out the DNA extraction and BeadChip validation analysis. TN, JV, AS, and NT conceived of the study, assisted in various aspects of data analysis, and provided critical commentary on the manuscript. All authors read and approved the final manuscript.

## Supplementary Material

Additional file 1 **Methylation validation using Sanger sequencing.** For validation of the methylation data from BeadChip, 17 genes were chosen, including unmethylated sites (*n* = 1), fully methylated sites (*n* = 2), and genes with tDMRs (*n* = 14) representing 36 CpG sites altogether. The x-axis shows DNA methylation beta-values obtained from BeadChip, and the y-axis shows beta values from Sanger sequencing. Click here for file

Additional file 2 **Global distribution of methylation.** The plot represents the methylation distribution of all specimens (70 samples) analyzed, as well as the controls of unmethylated (0%, negative control) and fully methylated (100%, positive control). The global distribution of methylated CpGs shows that most are either unmethylated or fully methylated in somatic tissues. Click here for file

Additional file 3 Description of tDMRs found by the simple linear model method of best fit according to MDL. Click here for file

Additional file 4 **Variance in tissues explained by gene regions and CGI regions.** (a) The figure is showing the distributions of the R squared statistic, which describes the variance explained by different gene regions and intergenic area. It is clear that gene body and intergenic areas are more variable than gene promoter areas. (b) Distribution of R squared statistic describes the variance explained by CpG island shores, shelves, and non-island regions. Figure shows, that CpG islands are the least variable among these groups. Click here for file

Additional file 5 GO analysis of hypermethylated tDMRs in specific tissue types. Click here for file

Additional file 6 **Hierarchical clustering of all the samples studied.** Hierarchical clustering of all the samples studied shows that the similarity between different tissues was much higher than between individuals, as tissues are mostly clustering together. Click here for file

Additional file 7 Gene expression datasets used for correlation analyses. Click here for file

Additional file 8**Correlation analysis of tDMRs and gene expression for methylations in the CGI-promoter and gene body regions.** (a) tDMR genes with low expression show high levels of methylation at CGI-promoter. (b) Gene body methylation in tDMRs is not correlated with gene expression. (a, b) The x-axis shows DNA methylation beta values, and the y-axis shows gene expression values. The different tissues studied are represented by the following symbols: aorta (•), coronary artery (●), bladder (), bone and joint cartilage (), bone marrow (), lymph node (), medulla oblongata (+), and tonsils (×).Click here for file

Additional file 9 PCR primers used in the methylation validation analysis. Click here for file

Additional file 10 **Correlations between consecutive probes.** Figure shows the correlation between methylation beta values of consecutive probes and how it depends on the distance between these probes. Click here for file
