# Erratum to: DNA methylome profiling of human tissues identifies global and tissue-specific methylation patterns

**DOI:** 10.1186/s13059-016-1091-0

**Published:** 2016-11-01

**Authors:** Kaie Lokk, Vijayachitra Modhukur, Balaji Rajashekar, Kaspar Märtens, Reedik Mägi, Raivo Kolde, Marina Koltšina, Torbjörn K. Nilsson, Jaak Vilo, Andres Salumets, Neeme Tönisson

**Affiliations:** 1 Institute of Molecular and Cell Biology, University of Tartu, Tartu, Estonia; 2 Department of Genetics, United Laboratories, Tartu University Hospital, Tartu, Estonia; 3 Estonian Biocentre, Tartu, Estonia; 4 Institute of Computer Science, University of Tartu, Tartu, Estonia; 5 Estonian Genome Center, University of Tartu, Tartu, Estonia; 6 Department of Medical Biosciences, Clinical Chemistry, Umeå University, Umeå, Sweden; 7 Competence Centre on Reproductive Medicine and Biology, Tartu, Estonia; 8 Department of Obstetrics and Gynecology, University of Tartu, Tartu, Estonia; 9 Institute of Bio- and Translational Medicine, University of Tartu, Tartu, Estonia

## Erratum

After the publication of this work [1] it was noticed that Additional file 1 is the same as Additional file 2.

Additional file 1 has now been correctly replaced.

## Additional file

Additional file 1: Methylation calidation using Sanger sequencing. For validation of the methylation data from BeadChip, 17 genes were chosen, including unmethylated sites (*n* = 1), fully methylated sites (*n* = 2), and genes with tDMRs (*n* = 14) representing 36 CpG sites altogether. The x-axis shows DNA methylation beta-values obtained from BeadChip, and the y-axis shows beta values from Sanger sequencing. (PDF 24 kb)
