# Supplementary figures and images for: DNA methylome profiling of human tissues identifies global and tissue-specific methylation patterns
Source: Genome Biol. 2014 Apr 1;15(4):r54. doi: 10.1186/gb-2014-15-4-r54 (PMC4053947; doi:10.1186/gb-2014-15-4-r54)

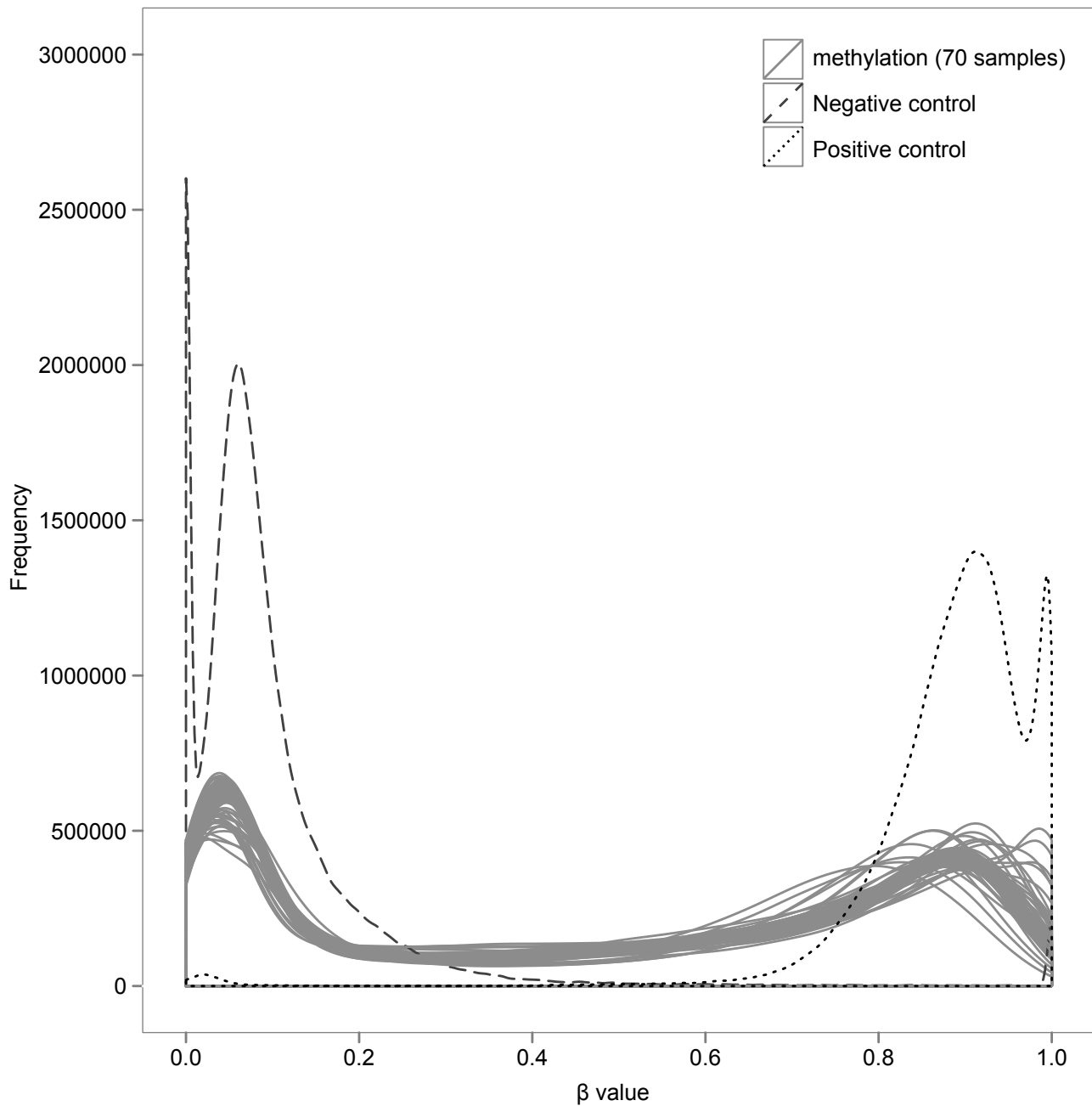

Supplement: Additional file 1 — Methylation validation using Sanger sequencing. For validation of the methylation data from BeadChip, 17 genes were chosen, including unmethylated sites (n = 1), fully methylated sites (n = 2), and genes with tDMRs (n = 14) representing 36 CpG sites altogether. The x-axis shows DNA methylation beta-values obtained from BeadChip, and the y-axis shows beta values from Sanger sequencing. [file gb-2014-15-4-r54-S1.pdf]

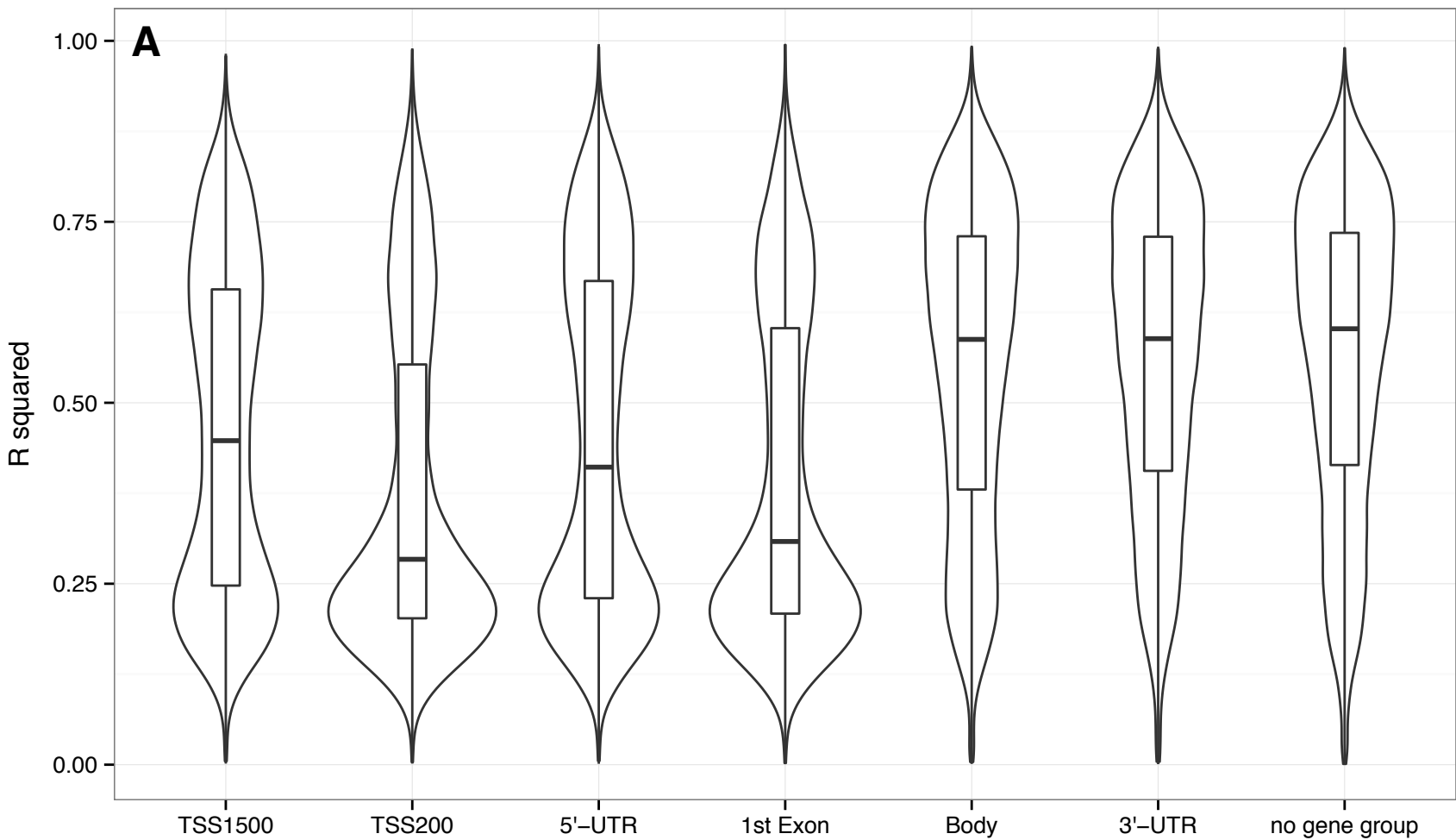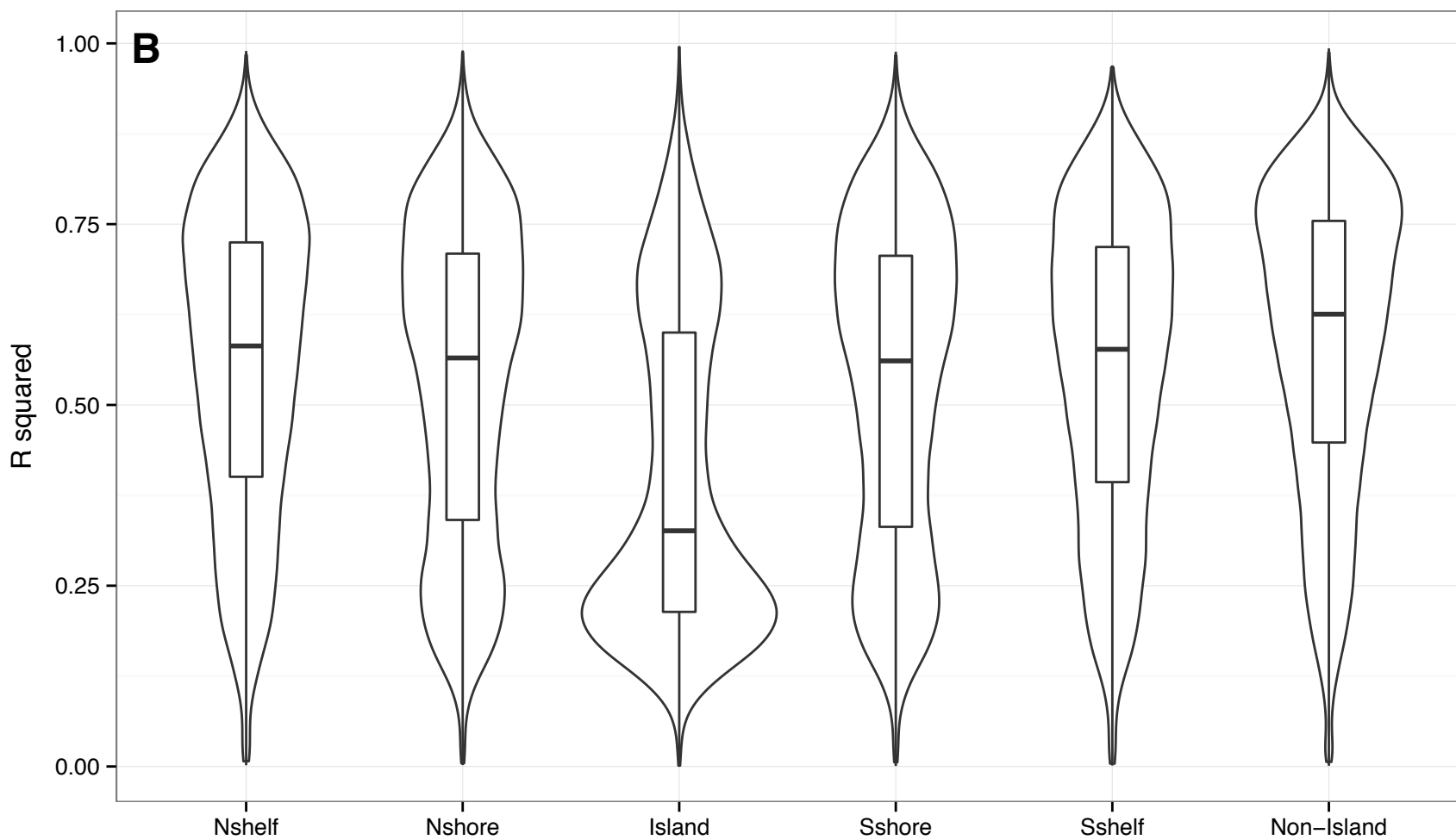

Supplement: Additional file 4 — Variance in tissues explained by gene regions and CGI regions. (a) The figure is showing the distributions of the R squared statistic, which describes the variance explained by different gene regions and intergenic area. It is clear that gene body and intergenic areas are more variable than gene promoter areas. (b) Distribution of R squared statistic describes the variance explained by CpG island shores, shelves, and non-island regions. Figure shows, that CpG islands are the least variable among these groups. [file gb-2014-15-4-r54-S4.pdf]

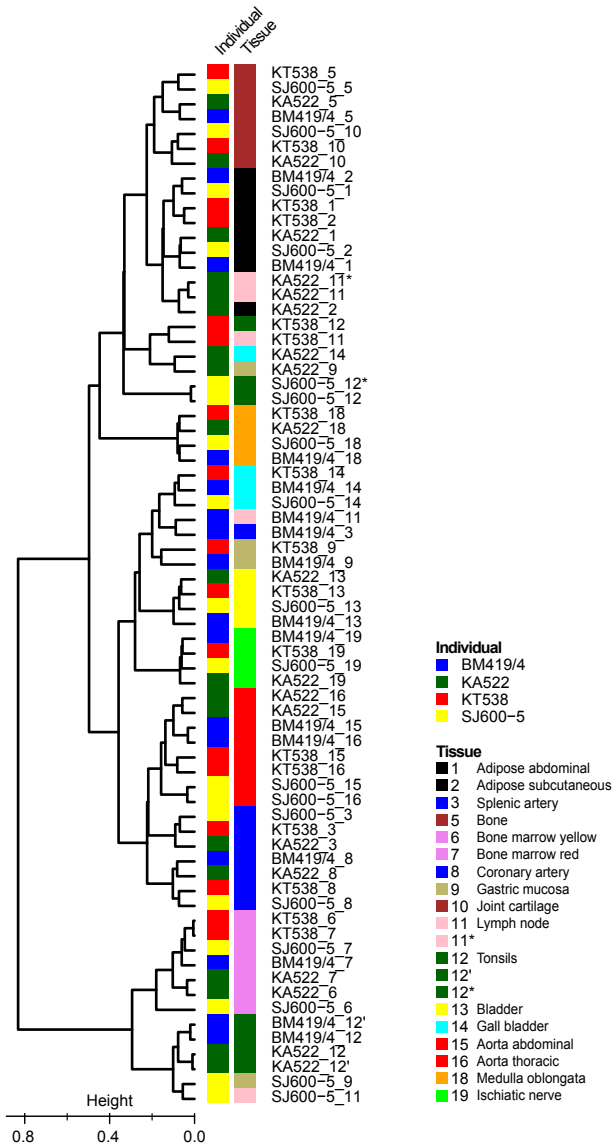

Supplement: Additional file 6 — Hierarchical clustering of all the samples studied. Hierarchical clustering of all the samples studied shows that the similarity between different tissues was much higher than between individuals, as tissues are mostly clustering together. [file gb-2014-15-4-r54-S6.pdf]

**A. tDMR promoter+CGI**

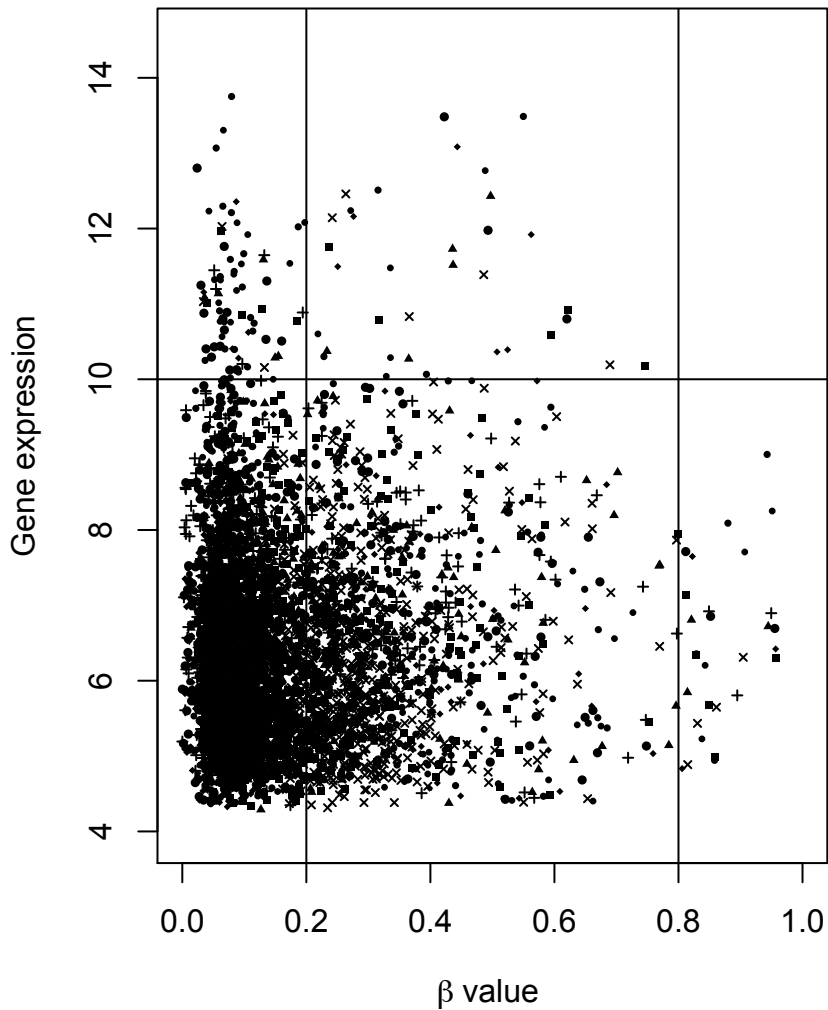

**B. tDMR body**

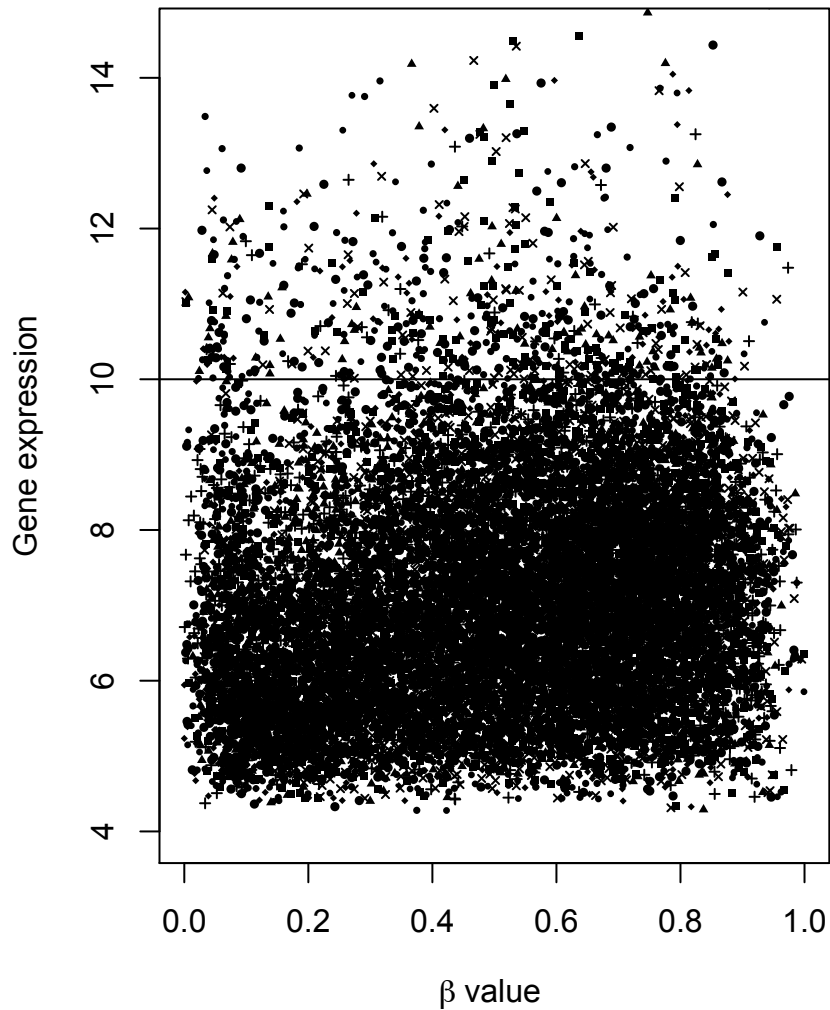

Supplement: Additional file 8 — Correlation analysis of tDMRs and gene expression for methylations in the CGI-promoter and gene body regions. (a) tDMR genes with low expression show high levels of methylation at CGI-promoter. (b) Gene body methylation in tDMRs is not correlated with gene expression. (a, b) The x-axis shows DNA methylation beta values, and the y-axis shows gene expression values. The different tissues studied are represented by the following symbols: aorta (•), coronary artery (●), bladder (), bone and joint cartilage (), bone marrow (), lymph node (), medulla oblongata (+), and tonsils (×). [file gb-2014-15-4-r54-S8.pdf]

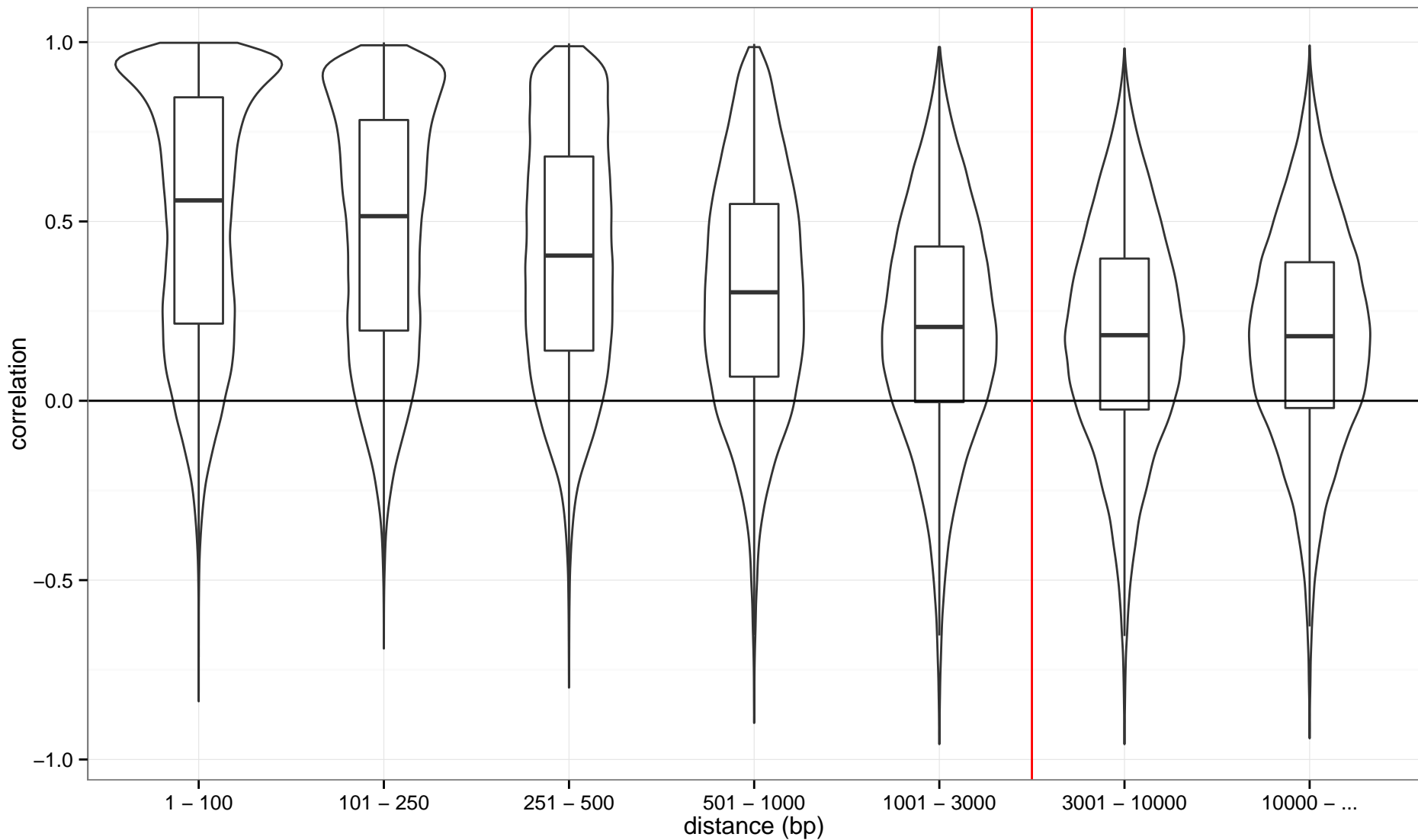

Supplement: Additional file 10 — Correlations between consecutive probes. Figure shows the correlation between methylation beta values of consecutive probes and how it depends on the distance between these probes. [file gb-2014-15-4-r54-S10.pdf]
