# Supplementary figures and images for: Erratum to: DNA methylome profiling of human tissues identifies global and tissue-specific methylation patterns
Source: Genome Biol. 2016 Nov 1;17:224. doi: 10.1186/s13059-016-1091-0 (PMC5090885; doi:10.1186/s13059-016-1091-0)

**SPN**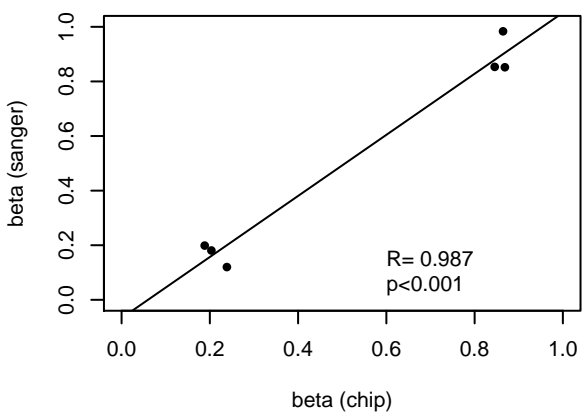**RUNX1**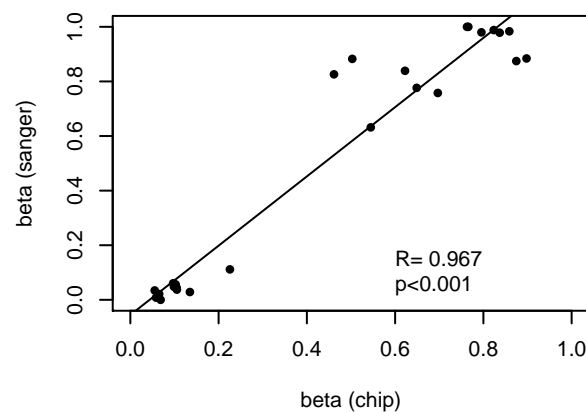**RHOH**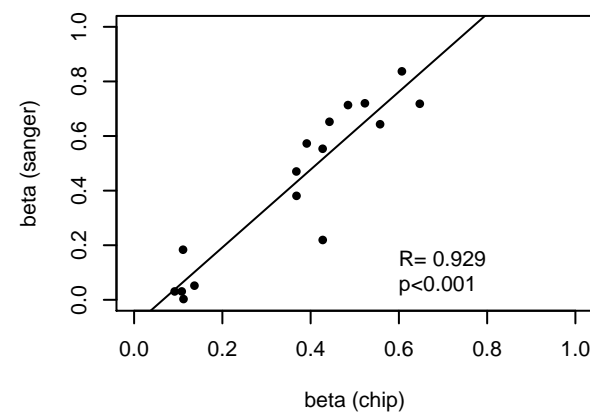**PTPN22**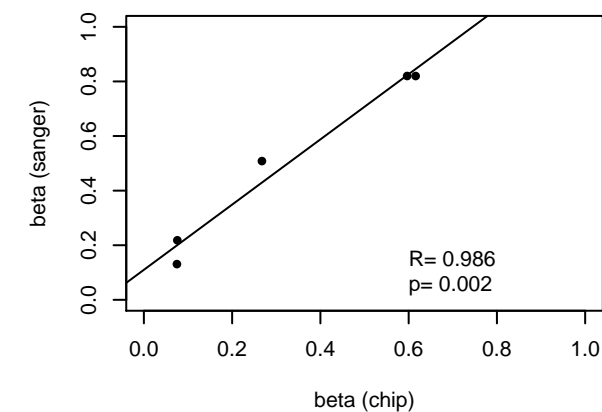**PRDM16**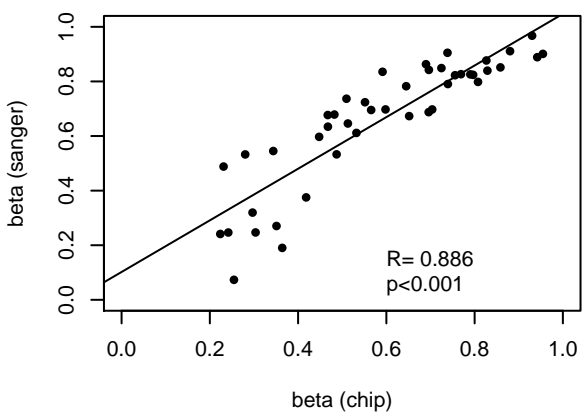**PLEK**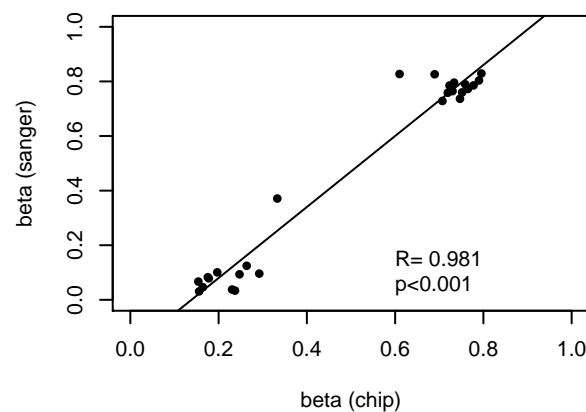**LMO2**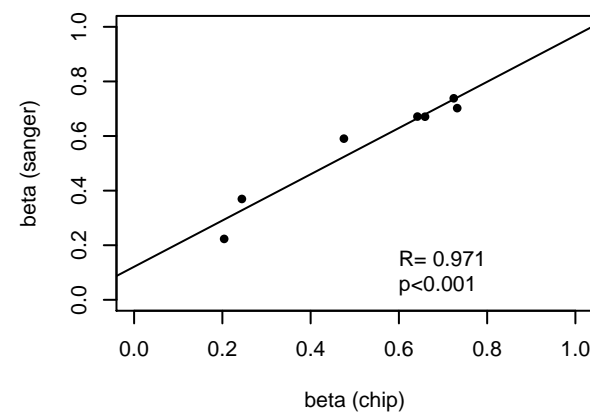**KIRREL3**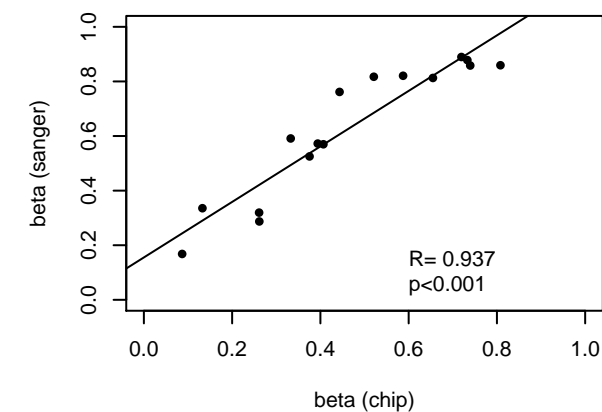**KALRN**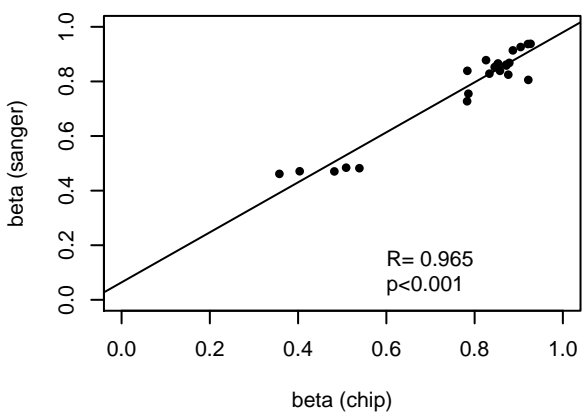**HCLS**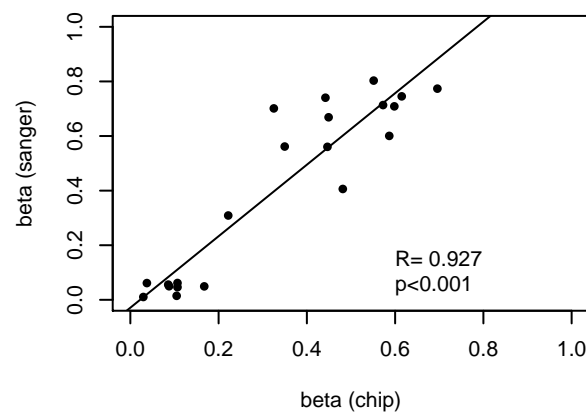**ENPEP**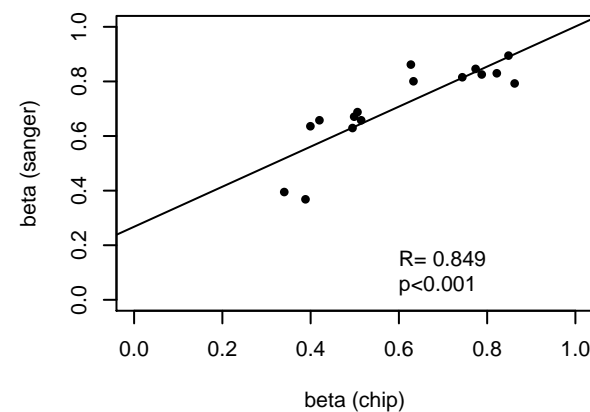**CPLX1**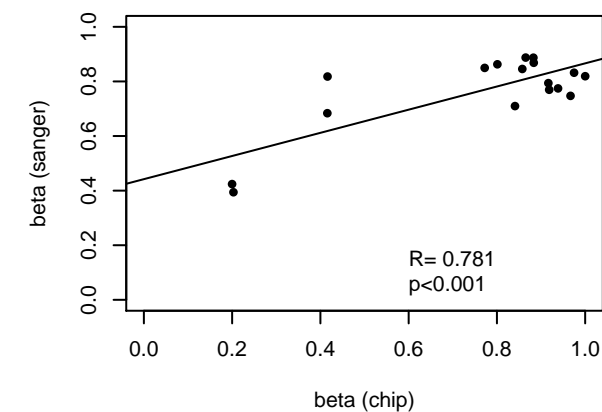**CDK6**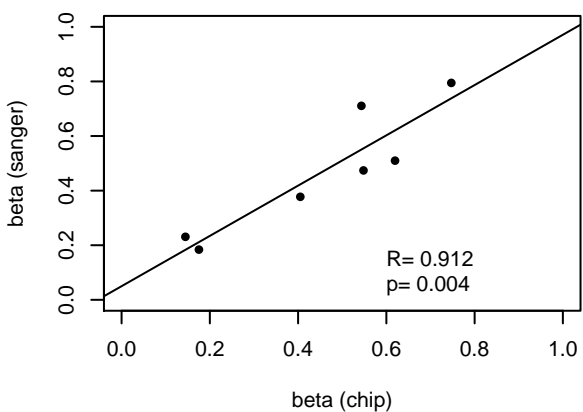**ANGPT2**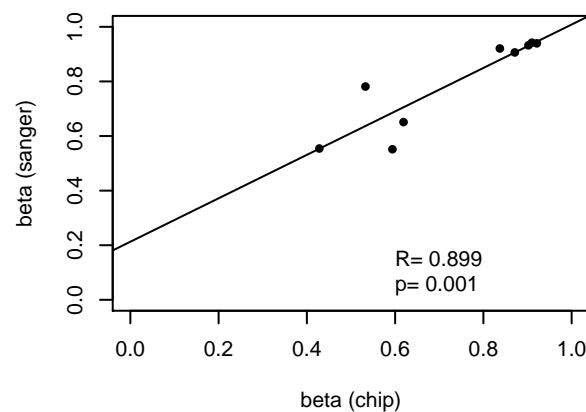**0% and 100% methylated CpGs**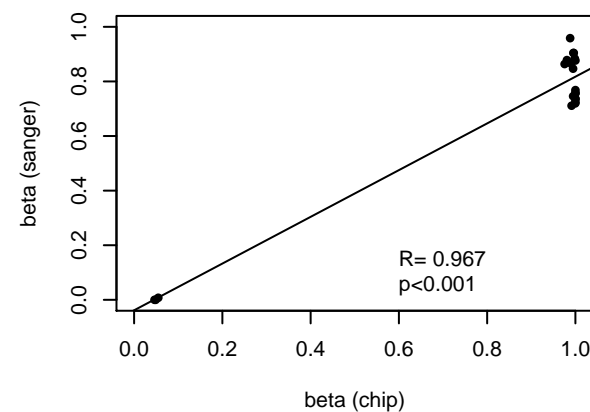

Supplement: Additional file 1: — Methylation calidation using Sanger sequencing. For validation of the methylation data from BeadChip, 17 genes were chosen, including unmethylated sites (n = 1), fully methylated sites (n = 2), and genes with tDMRs (n = 14) representing 36 CpG sites altogether. The x-axis shows DNA methylation beta-values obtained from BeadChip, and the y-axis shows beta values from Sanger sequencing. (PDF 24 kb) [file 13059_2016_1091_MOESM1_ESM.pdf]
